# Universal Cold Snare Polypectomy for Small and Diminutive Colonic Polyps—Sustainability Matters

**DOI:** 10.1111/den.70150

**Published:** 2026-03-31

**Authors:** Anjan Dhar

**Affiliations:** ^1^ Department of Gastroenterology Darlington Memorial Hospital & Teesside University Middlesbrough UK

1

Gastrointestinal endoscopy continues to be the third highest generator of greenhouse gases (GHGs) amongst medical specialities, after anesthesia and intensive care and every year, over 22,000,000 endoscopies are undertaken in the United States and approximately 1.5 million in the United Kingdom [1, 2]. A previous study estimated that, on average, just one endoscopic procedure generates approximately 2.1 kg of waste which is roughly the same as the total waste generated by an individual person in the United States in one day [3]. There are multiple streams of waste generation during an endoscopic procedure which include automated reprocessing of reusable endoscopes, electricity, water, chemicals, consumables and accessories, and computers as well as printers. The impact of greenhouse gases on planetary health has prompted national and international endoscopic societies to produce guidelines for the reduction of waste generated during endoscopy as well as the drive toward recycling of consumables and accessories [4, 5].

The European Society of Gastrointestinal Endoscopy (ESGE) in its 2024 update to Colorectal polypectomy and endoscopic mucosal resection guideline recommends the resection of all polyps with the exception of diminutive (≤ 5 mm) rectosigmoid polyps that are predicted to be non‐adenomatous with high confidence. Furthermore it recommends cold snare polypectomy for the removal of diminutive polyps (≤ 5 mm) including a clear margin of normal tissue (1–2 mm) surrounding the polyp. It also recommends against the use of cold and hot biopsy forceps excision because of its high rate of incomplete resection, and deep thermal injury with the hot biopsy forceps. Small polyps (6–9 mm) should be removed by cold snare polypectomy with a clear margin of tissue (1–2 mm) surrounding the polyp. Hot snare polypectomy for small polyps is not recommended [6]. The environmental impact of cold snare polypectomy compared with hot snare polypectomy for diminutive and small polyps has not been assessed. Besides the direct greenhouse gas impact of the cold snare compared with the hot snare, other factors such as the indirect greenhouse gas impact of the diathermy generator, and the disposal of the patient plate are expected to make hot snare polypectomy less environmentally friendly. Furthermore, the incidence of adverse events, post polypectomy complications and consequent hospital admissions as well as additional procedures need to be taken into account for a complete Lifecycle assessment of polypectomy. The ESGE‐ESGENA Position Statement paper on reducing the environmental footprint of gastrointestinal endoscopy also recommends a rational use of endoscopic accessories during the procedure (Statement 8) and also favors the use of cold snare polypectomy and under water endoscopic mucosal resection in validated indications to reduce the carbon footprint [7].

In this journal, Hao‐Yu et al. report the carbon footprint of a universal cold snare polypectomy approach for diminutive and small polyps compared to a legacy forceps biopsy (for diminutive polyps) and hot snare polypectomy (for small polyps) at their institution over a 12‐month period. It is interesting to note that the authors were still using forceps and hot snare polypectomy for resection of both diminutive and small polyps at their institution which may not be the case in the Western world. Reassuringly, and quite understandably, the universal cold snare polypectomy strategy resulted in an approximately 5% reduction in GHG emissions per colonoscopy, compared with their legacy approach. This reduction is driven by the three times higher carbon footprint of post polypectomy complications arising from the forceps plus hot snare approach—this makes it obvious as to why the use of forceps and hot snares for small polyps is no longer recommended by most learned societies. The reduction also remains consistent across Fecal Immunochemical Test (FIT) positive cohort and the post polypectomy surveillance cohort. The authors do not mention if polypectomy specimens from the same colonic segment were put into a single specimen pot or in multiple pots, to reduce carbon footprint of histology.

It is important to recognize the real magnitude of GHG impact arising from the use of cold snare polypectomy in relation to the total number of polypectomies that might be expected to be carried out in an average endoscopy unit. Analysis from a large tertiary academic endoscopy unit in the UK reported that 89% of all polyps resected in a 12‐month period were between 0 and 10 mm in size [8]. One also needs to recognize that colonoscopists need to be meticulous and have a good resection technique for CSP, in keeping with EGSE guidelines, to have a clear margin of 1–2 mm of normal colonic mucosa around the resected margin. This reduces the risk of residual adenoma and recurrences at subsequent colonoscopy and also the risk of post‐colonoscopy colorectal cancer (PCCRC) arising from incompletely resected adenomas. The GHG emission related to CSP reported in this paper is based on the “no pre‐injection” approach where the small adenoma or sessile serrated lesion is not lifted using a lifting solution. The use of an injection needle and lifting solution prior to CSP will increase the GHG emission related to the procedure. Furthermore, these data related to the universal use of CSP for small and diminutive polyps only and cannot be extrapolated to larger polyps of 10–19 or > 20 mm. This is because the residual adenoma rates and recurrences from the use of CSP technique for larger polyps have been reported to be higher than hot snare polypectomy and therefore the benefit of GHG emissions might be off‐set by the impact of adverse events. Other factors that influence the GHG emissions related to polypectomy technique and type of snare used include anti‐thrombotic medication use during colonoscopy and post‐polypectomy complications of bleeding, pain, hospital readmissions, repeat procedures, residual adenoma and cancer development.

However, a crucial dimension of clinical sustainability is also “systems sustainability,” which refers to change not just at an individual level but also at an organizational and national level. This requires the sustainability impact of universal cold snare polypectomy for small and diminutive polyps to be cascaded to all colonoscopists in an organization and also to trainees from the very beginning, to “get it right first time (GIRFT).” National endoscopy databases should record not just the procedure of polypectomy but also how it was done (HSP v CSP) and whether injection and clipping were also used. This data can then be audited to implement sustainable practices both at the individual and organizational level.

In summary, as the healthcare sector grapples with increasing demands of care, with finite resources, sustainable clinical practice is imperative to reduce the carbon footprint of what we do. All colonoscopists should be encouraged to adopt environmentally sustainable strategies in their colonoscopy and polypectomy practice, and wherever possible, maximize the rational use of accessories (injection, snares, clips, etc.), thereby minimizing the harm to planetary health. The use of universal cold snare polypectomy without prior injection lifting should be standard practice for small and diminutive colonic polyps.

## Funding

The author has nothing to report.

## Conflicts of Interest

The author declares no conflicts of interest.

## Linked Article

This article is linked with K. Takabayashi, *Journal of Gastroenterology* (2025). http://doi.org/10.1111/den.70150.
